# Integrating patient- and caregiver-reported outcome measures into the daily care routines of specialised outpatient palliative care: a qualitative study (ELSAH) on feasibility, acceptability and appropriateness

**DOI:** 10.1186/s12904-022-00944-1

**Published:** 2022-05-02

**Authors:** Hannah Seipp, Jörg Haasenritter, Michaela Hach, Dorothée Becker, Dania Schütze, Jennifer Engler, Cornelia Ploeger, Stefan Bösner, Katrin Kuss

**Affiliations:** 1 Department of General Practice and Family Medicine, Philipps-University of Marburg, Karl-von-Frisch-Straße 4, 35032 Marburg, Germany; 2 Professional Association of Specialised Palliative Homecare in Hesse, Weihergasse 15, 65203 Wiesbaden, Germany; 3 Institute of General Practice, Goethe-University Frankfurt, Theodor-Stern-Kai 7, 60590 Frankfurt am Main, Germany

**Keywords:** Palliative Care [MeSH], Home Care Services [MeSH], Quality of Health Care [MeSH], Needs Assessment [MeSH], Qualitative Research [MeSH], Patient Outcome Assessment [MeSH], Patient Reported Outcome Measures [MeSH], Routinely Collected Health Data [MeSH]

## Abstract

**Background:**

The use of patient-reported outcome measures (PROM) and caregiver-reported outcome measures can raise the patient centeredness of treatment and improve the quality of palliative care. Nevertheless, the everyday implementation of self-report in patients and caregivers is complex, and should be adapted for use in specific settings. We aimed to implement a set of outcome measures that included patient and caregiver self- and proxy-reported outcome measures in specialised outpatient palliative care (SOPC). In this study, we explore how the Integrated Palliative Outcome Scale (IPOS), IPOS Views on Care (IPOS VoC) and the Short-form Zarit Caregiver Burden Interview (ZBI-7) can be feasibly, acceptably and appropriately implemented in the daily care routines of SOPC.

**Methods:**

Five SOPC teams were trained, and used the outcome measures in daily practice. Team members were mainly nurses and physicians. To investigate their feedback, we used a multi-method qualitative design consisting of focus groups with SOPC-team members (*n* = 14), field notes of meetings and conversations with the SOPC teams. In an iterative process, we analysed the findings using qualitative content analysis and refined use of the outcome measures.

**Results:**

We found that integrating patient and caregiver outcome measures into daily care routines in SOPC is feasible. To improve feasibility, acceptability and appropriateness, the resulting burden on patients and relatives should be kept to a minimum, the usefulness of the measures must be understood, they should be used considerately, and administration must be manageable. We removed ZBI-7 from the set of measures as a result of feedback on its content and wording.

**Conclusions:**

SOPC-team members have reservations about the implementation of PROM in SOPC, but with appropriate adjustments, its application in daily care is feasible, accepted and perceived as appropriate. Previous to use, SOPC-team members should be trained in how to apply the measures, in the design of manageable processes that include integration into electronic documentation systems, and in ongoing evaluation and support. They should also be taught how useful the measures can be.

**Trial registration:**

May 19^th^, 2017, German Clinical Trials Register DRKS-ID: DRKS00012421.

**Supplementary Information:**

The online version contains supplementary material available at 10.1186/s12904-022-00944-1.

## Background

In Germany, just as in other countries, specialised outpatient palliative care (SOPC) is available for patients with life-limiting diseases and complex needs, who would prefer to receive treatment in an outpatient setting [1]. Multidisciplinary teams care for patients at their places of residence, e.g. at their homes, in order to promote patients’ self-determination and quality of life [2]. SOPC teams provide comprehensive care, including physical and psychosocial care, and also take relatives into account, who themselves often provide informal care.

Patient-reported outcome measures (PROM) and caregiver-reported outcome measures have proved their worth in palliative care and they are being increasingly used in practice [3]. On a patient level, they help identify and address patients’ unmet needs [4], while on a provider level, their use permits case-by-case evaluation of care, and on a policy level, they allow care to be monitored [5]. Previous research has shown that the implementation of patient-reported outcome measures (PROM) is complex and needs to be adapted to the specific setting in which it is used [6, 7].

To take into consideration these complex needs, there are multiple frameworks that aim to support this work. The Consolidated Framework for Implementation Research (CFIR) implies that beyond the characteristics of the intervention, also the inner and outer setting, the implementation process as well as individuals influence successful implementation. [8] Proctor et al. emphasize that the exploration of implementation outcomes is key to understand how implementation can be successful. They describe conceptually distinct outcomes, which can be relevant in the evaluation of an implementation. They include, among others, the stakeholders’ view on acceptability, appropriateness and feasibility [9].

The study presented in this paper is part of the ELSAH-study (‘Evaluation of Specialised Outpatient Palliative Care by taking the example of Hesse’). The aim of ELSAH is to ensure that all SOPC teams in Hesse, a federal state in Germany, implement measures that enable the evaluation of quality of care in SOPC (work package I) by focusing on outcome measures from the perspective of patients and relatives [10]. Data collected before our study were based on the National Hospice and Palliative Care Registry and comprise mainly data on structure and process quality, symptoms, and treatment and support needs from the perspective of health professionals. Patients’ and relatives’ perspectives were barely considered at all [11]. We are striving to integrate tools into existing documentation and analysis structures to ensure their sustainable implementation and use. The study has already enabled us to identify topics that contribute to providing successful care from the perspectives of those involved [12]. Based on these findings and an overview of the literature on outcome measures used in palliative care, we have put together validated tools to a set of measures [10] that are designed for use in a palliative care setting and that can help support the team in their work [13]. The set of measures is based on the Outcome Assessment and Complexity Collaborative (OACC) suite of measures [14] and includes, amongst others, the Integrated Palliative Outcome Scale (IPOS) [15], and IPOS Views on Care (IPOS VoC) [16]. As a previous phase of the study showed that family members play a central role, we have further included the Short-form Zarit Caregiver Burden Interview (ZBI-7), which is also recommended for use in palliative care [17].

A further study has highlighted patients’ appreciation of the use of IPOS in a SOPC setting on the basis that it resulted in the adaptation of care to meet their needs [18]. Another study has examined the views of patients, informal caregivers and health professionals on the use of the OACC suite of measures across different palliative care settings [13]. The authors of that study recommend its stepwise implementation under consideration of the available organisational infrastructure, the teams’ motivation, the rationale for use, and training in the skills required to apply such tools in practice. The findings of further studies that the implementation of the targeted outcome measures is feasible in a specialised palliative care setting for inpatients cannot automatically be transferred to the SOPC setting, as the needs of patients, the organisation of care, and the extent of family involvement, all vary in this setting [19–21].

We intend to establish the implementation of such outcome measures in all Hessian teams for use in routine care, and to publish our results elsewhere. For this small-scale implementation study before the larger rollout in all hessian SOPC-teams, we followed proctor’s taxonomy of three implementation outcomes and applied the them to our study as follows [9]:

1. Feasibility: Extend to which the use of the outcome measures is feasible in SOPC.
2. Acceptability: Perception among SOPC team members that the use of the outcome measures is agreeable, or satisfactory.
3. Appropriateness: Perceived fit or relevance of the use of the outcome measures in SOPC for use in daily work and for presenting the quality of care.

## Methods

### Aim

The aim of this study was to explore whether implementing the IPOS, IPOS VoC and ZBI-7 outcome measures in the daily care routines of SOPC is feasible, acceptable and appropriate.

### Setting

The study took place in Hesse, a federal state of Germany with about 6.3 million inhabitants. Adult German SOPC-patients mostly suffered from cancer [22]. SOPC can be prescribed by outpatient physicians or hospital physicians [23]. SOPC team members are physicians, nurses and sometimes social workers and psychologists. All 25 SOPC teams in Hesse are members of the Hessian Professional Association of Specialised Palliative Homecare and have a common strategy of collecting standardised data and analysing them regularly in order to improve quality [24, 25]. The teams use electronic documentation systems (EDS) for data collection, but not all from the same software provider.

### Sampling

We included five of the SOPC teams that provide care to adults in the state of Hesse. We purposively sampled the SOPC teams for team location (rural and urban locations), and the use of differing EDS, to address software-related issues with regard to the expansion in all hessian teams. The SOPC teams knew the research team from a previous phase of the study [12].

### Design

In line with Proctor's recommendations to explore these implementation outcomes, we used a qualitative design including the direct involvement of stakeholders [9]. The multi-method qualitative design included focus group discussions, the researchers’ written field notes of all conversations and meetings with the SOPC teams, as well as field notes of feedback from SOPC-team members to the research team about the use of the measures (face-to-face, per telephone, or per e-mail). To examine integration into daily care, we applied an iterative process, which included testing their use in SOPC, collecting feedback from health professionals, adjusting the process depending on the feedback, and examining the effect of the adjustments [26].

Table 1 shows the outcome measures we used in this study, including content, target population and respondents, as well as information on when and which versions were used.

**Table 1:** Patient reported outcome measures used in this study

**Measures**	**Integrated Palliative care Outcome Scale (IPOS) **[15]	**IPOS Views on Care (IPOS VoC) **[16, 28]	**Zarit Caregiver Burden Interviews (ZBI-7),** **7-Item Version **[17, 29]
Target population	Patients	Patients	Informal caregivers
Content	Ten questions covering the main problems, including physical, psychological, and spiritual problems, and practical concerns	Four questions on quality of life, and requesting an evaluation of the influence of the palliative care team on the current situation	Seven questions on the burden of care on informal caregivers
Respondents	Patient self-reporting or proxy-reporting by relatives or staff	Patient self-report	Relative self-report
When the measures were used	On admission, after about 5–10 days, and at ≥ 3 further appointments; at least once during further care, and when changes occurred	On admission, after about 5–10 days, and at ≥ 3 further appointments; at least once during further care and when changes occurred	On admission; at least once during further care
Version	Validated German Versions [30]	Own translation into German	7-item version recommended for palliative care; translation into German based on a German version validated for dementia care [31]

#### a) Initiation: preparation and team training

We started the implementation in one team and gradually added the other four. We presented and discussed the study plan in preparatory meetings (JH, HS, KK) with members of two of the SOPC teams. All team members were invited to participate in training sessions (held by HS, KK) at the teams’ offices. We explained the different measures to the teams, and talked about content, aims, how to use them with the respondents in practice and benefits of the outcome measures in practical care. We gave the teams case folders containing information on the measures. We started using the paper-based version in order to be able to adjust the case folder flexibly, and to allow for written comments. We collected the changes and entered them into the existing electronic documentation system (EDS) at a later date to prevent unnecessary programming. The teams started using the measures in daily practice immediately after the training meeting.

#### b) Application in daily practice and support

SOPC-team members handed the paper-based measures to patients and their informal caregivers and asked them to complete the forms either alone, or with the assistance of a SOPC-team member. Afterwards, they collected the completed forms. We encouraged the SOPC-team members to contact the study team in case of questions, comments, and problems arising from the use of the measures. Evaluation meetings were arranged when there was a need to discuss matters face-to-face. We wrote field notes on all telephone calls and meetings for preparation, training and evaluation. Field notes were taken of the date, team, type of contact, content, and the researchers’ comments [27].

#### c) Evaluation: focus groups

Two of the three researchers (HS, KK, JH) conducted focus groups with two of the SOPC teams to gain insight into their experiences and to collect suggestions on the use of the tools (Additional file 1: A: focus group topic guide) [32]. We wrote field notes during the focus groups, and transcribed relevant passages of the audio files verbatim [33].

### Analysis

To analyse feedback from the health professionals, we used an iterative process for qualitative content analysis [34, 35]. For this purpose, we imported all field notes and audio files from the focus groups and entered them into MAXQDA 2018 software. We also triangulated all field notes and focus group data [36, 37]. We predefined codes according to our research interest and prior knowledge from data collection, and we added inductive codes for topics emerging from the data [35]. We (HS, KK, JH) discussed all emerging topics at all stages of data collection and identified those that were relevant. We adjusted the process accordingly and discussed major decisions at conferences with the whole study group until we reached a consensus.

### Ethics, data protection

All SOPC teams represented by the Professional Association of Specialised Palliative Homecare in Hesse agreed to participate in the ELSAH-study. Participants in focus groups gave their written informed consent for audio recording before they began. SOPC-team members obtained patients’ and caregivers’ written consent to complete the measures for research purposes. The study was approved by the Ethics Committee of the Faculty of Medicine, Philipps University Marburg (05–04-2018; ref. 47/18).

## Results

### Sample

#### Participating SOPC-teams

All five invited SOPC teams agreed to participate, and all team members applied the outcome measures. The teams all had about 20 members of staff, of which about 60% are nurses and 40% physicians. Four teams started by using the paper version and switched to the software version later. The fifth team started using the software version directly. Four teams used the same EDS, which about 80% of the hessian SOPC-teams use. One team used another EDS. We met twice for preparation, eight times for training and four times for evaluation purposes. Table 2 presents the course of the study from April 2018 to January 2019 for each team.

**Table 2:** Timeline of study

	**Team 1 (urban)**	**Team 2 (rural)**	**Team 3 (urban)**	**Team 4 (rural)**	**Team 5 (urban)**
April 2018		Preparation			
May	Training/Start (P)				
June		Training/Start (P)		Preparation	
July	Evaluation		Training/Start (P)		
August	Focus group	Evaluation	Evaluation	Training/Start (P)	
September					
October		Evaluation	Focus group		
November		Training (S)	Training (S)		Training/Start (S)
December				Evaluation	
January 2019	Training (S)				

*Training (P)* Training beginning with paper-based version, *Training (S)* Training beginning with software version

#### Focus groups

The two focus groups took place at the SOPC teams’ offices. We invited all team members of Team 1 and Team 3 to participate, but some did not work that day or work-related matters prevented their participation. Table 3 shows characteristics of the focus groups and their participants.

**Table 3:** Characteristics of focus group participants

		**Focus group 1** **(Team 1)**	**Focus group 2 (Team 3)**	**Total**
Number of participants; n		7	7	14
Duration; minutes		120	36	-
Gender; n (%)	Female	6 (85.7)	4 (57.1)	10 (71.4)
	Male	1 (14.3)	3 (42.9)	4 (28.6)
Age; years	Mean (Min, Max)	48.4 (34, 61)	40.1 (27, 52)	44.3 (37, 61)
Profession; n (%)	SOPC nurse	6 (85.7)	5 (71.4)	11 (78.6)
	SOPC coordinator	1 (14.3)	0	1 (7.1)
	SOPC physician	0	1 (14.3)	1 (7.1)
	SOPC social worker	0	1 (14.3)	1 (7.1)
Work experience in SOPC; years	Mean (SD)	3.3 (1.4)	5.9 (4.7)	4.6 (3.6)

*SD* standard deviation

### Feasible, acceptable and appropriate use of the measures

We identified problems in the overall usage of the outcome measures and in the use of specific measures, but found solutions and refined the process accordingly. We present each issue below, and provide feedback on particular measures. We illustrate our findings with pseudonymised quotations from the focus groups, which we have translated into English. Issues arose for all methods of data collection, but not necessarily for all participants.

#### Avoid overburdening patients and relatives

Acceptability and appropriateness were limited, when SOPC-team members feared to burden patients or relatives and to harm the quality of relationship by the use of the measures. Most SOPC-team members said they feared overburdening patients and relatives by asking them to complete forms and discuss sensitive topics, which was something they thought might be an additional and unreasonable burden in the palliative situation.

*I think that for OUR patients - both for patients and their relatives – it’s often asking too much of them. They find themselves in a life-limiting situation, they are often completely stressed out, especially the relatives, so that it’s difficult for ME to give them something extra on top … that they have to fill out [...]. My personal opinion is that if our patients are doing well enough to occupy themselves with such a questionnaire, then I would rather they spend their time differently, use it for something else, namely with each other. (1728E, female nurse)*

Some health professionals were afraid of weakening their relationships with patients and relatives by allowing the outcome measures to dictate the care they provided, rather than focussing on what the patients actually required. Others, on the other hand, saw advantages in using the measures, and promoted their use.

#### Understanding their usefulness

All types of data collection showed that understanding how useful the measures are is key to being accepted and to be perceived appropriate by SOPC-team members. Some team members doubted the need to measure quality out of principle on the grounds that the patients provided them with direct feedback on the quality of care anyway. Others said they could not imagine how use of the outcome measures could result in improvements to care.

*They’re all issues that are familiar to us! Why should we document it all? We do it, and we enter the information into [our documentation software]. But why? Why do we have to do all that as well? (1722E, female nurse)*

Some participants had reservations about the validity of the assessments. They argued that they could be biased by the fact that patients were dependent on their SOPC teams and suggested asking bereaved relatives instead. Some SOPC-team members further presumed that analyses of aggregated data would be biased because assessments were not always obligatory, on the assumption that some SOPC-team members would skip assessments they considered unnecessary. It became obvious in a focus group that several SOPC-team members used the measures because they felt they were required to, but that they took no further interest in the results.

Some SOPC-team members raised the question whether it was really possible to measure quality of care in this way because patients’ health generally deteriorated until they ultimately died. Participants further feared that it would be impossible to show any development in care, especially in cases of short duration.

*Can you arrive at a correct result by doing this? [...] The situation of our patients won’t, won’t get any better. It will get worse and worse and worse and then you won't get any positive answers. (1727E, female nurse)*

SOPC-team members were afraid that misinterpretation of data could lead to harassment, or be used to force them to provide care in a specific manner.

*What I, personally, am really worried about is that we get modules, and we are told what to do with our time: ‘Home visits shouldn’t take more than half an hour. You can get rid of this and get rid of that.’ No, you can't! And then we won't talk about ´SOPC´ anymore, or about quality. (1723E, female nurse)*

Participants also wanted to understand the usefulness of the outcome measures and suspected that this would increase their motivation to use them.

*I want to understand it. And I’d like to feel convinced that it is something that it makes sense to participate in on the basis of my own understanding that it could work. (1722E, female nurse)*

We therefore revised the training to focus more on usage and usefulness. One topic in these meetings was the potential for improvement that the outcome measures offered in individual cases, and their usefulness in daily practice, for example in visualising care and communication processes in a team. We further discussed the importance of measuring quality in the healthcare system, and spoke about the weight attached to data sovereignty to reduce fears of misuse. The use of aggregated data to achieve internal quality improvements and to help explain quality of care to external audiences were further topics of the meeting.

#### Enable sensitive use

Sensitive use promoted feasible use and strengthened acceptance among SOPC-team members. Some SOPC-team members felt uncomfortable handing out questionnaires and reading out the items word-for-word. In their opinion, some topics should be adapted to each individual, with use of the questionnaires generally requiring empathy. For the same reason, they thought it was wrong to use them at a predefined time point.

*The other thing is that things happen when it’s their turn to happen. My problem is that when I feel as though; when I continue talking and touch a sore spot, then I have reached a point when it’s time to stop [the survey]. And I have to work that out myself. No questionnaire can judge that. (1723E, female nurse)*

To ensure the survey is used with respect for sensitive topics, we encouraged SOPC-team members to integrate the patients’ self-reported views into conversations, to abstain from using the question’s exact wording if necessary, thus relying more on the patient’s narrative. Participants described this as being difficult to begin with, but added that it became easier over time.

*So, when I have filled out the form like that [in conversation], then I generally did it by devoting part of the conversation to the questions, but what I never did was to read them out loud, so to speak, and use the exact wording, you see? [...] Then things went ok. (1729E, male physician)*

We also left it to the professionals to decide if and when it was reasonable to broach a specific topic at a certain time. We therefore avoided arranging predefined time points for self-report, but instead made the default to ultimately assess every topic when the information was collectible, at the very least in the form of a proxy-report. Topics that are relevant in a particular case should nonetheless be reviewed regularly, even when the situation changes, for example because of deterioration in health. Participants said this was feasible for them:

*When we’ve built up a relationship of trust in the course of our work, then when we strike up a conversation, it sometimes happens in passing that you end up being able to tick a box. [...] That is the way to do it. (1729E, male physician)*

#### Manageable administration

Manageable administration was key to a feasible implementation and influenced the acceptance of use. Most SOPC-team members said that handing out paper-based forms to the patients and relatives, explaining and asking them to fill them out, collecting them afterwards, and analysing the results was a major effort:

*And before every home visit you have to think about it: Ah yes, there’s a questionnaire of the patient’s, what do I have to take with me? (1727E, female nurse)*

It became clear that previously planned integration into the electronic documentation system was necessary because SOPC-team members considered it more practical:

*If I have this question in my [documentation software] and I answer it then, when it’s relevant [...] then everything’s ok and I can do it. But not when I have to it at a specific time (1723E, female nurse)*

SOPC-team members further outlined that self-report was not possible for all patients. The reasons they described for non-participation were health deterioration, language barriers, and cognitive and psychological impairment. Participants said that short periods of care resulting from, for example, death, or a change in the place care is provided, complicate the use of measurement tools.

*It struck me that we had […] a lot of patients suffering from dementia, or who were so weak, ill, or whatever, and close to death that it was not really possible to do more than fill out the symptoms via proxy-report. I noticed that we were very often not in a position to answer interesting questions like ‘What has been worrying you?’ (1729E, male physician)*

SOPC-team members appreciated the involvement of relatives when measuring outcomes, as they are also affected. Nevertheless, they said that relatives could not always make assessments because of language barriers, cognitive and psychological impairment, or because they were not involved in providing care. SOPC-team members also said they could not provide information on all topics via proxy-reports, pointing out that while they are able to assess some topics such as physical condition, they considered it presumptuous to comment on psychosocial subjects such as ‘quality of life’:

*I don’t think proxy-reporting is really possible at all. I cannot presume to judge how someone felt three days before we took part in care. (1728E, female nurse)*

In consequence, we encouraged SOPC-team members to handle the items more flexibly and to alternate between self- and proxy-reporting. Patient-reported outcomes remained our first choice, but if it was not possible or reasonable, the measures could be still assessed by relatives acting as proxies (second choice), or health professionals as a third possibility. When SOPC-team members said they were unable to answer, we provided the response option ‘not assessable’ for every item of the proxy-report version.

SOPC-team members said patients, relatives and health professionals sometimes differed in their views. They considered this as interesting from a care perspective, so we included a marker to indicate who had reported the item in the electronic documentation.

#### Feedback on specific measures

SOPC-team members confirmed that the content of the measures was relevant to the topics of care under investigation. IPOS’ main problems and symptoms were accepted and perceived appropriate and feasible, but the formulations of the other items were considered inadequate and difficult to understand. SOPC-team members were divided over if IPOS VoC was appropriate and acceptable, but most reported of limited feasibility. ZBI-7 was neither assessed appropriate nor acceptable. Detailed feedback and adaptions relating to the specific measures are described in Table 4.

**Table 4:** Feedback and adaptation of measures used

**Measures**	**Integrated Palliative care Outcome Scale (IPOS)** [15]	**IPOS Views on Care (VoC)** [16, 28]	**Zarit Burden Interview (ZBI)** [17]
Feedback relating to the measure	The HPs said that IPOS covered relevant topics of care, and that when patients were able to complete a written questionnaire, filling in the self-report form did not cause problems The HPs explained that questions on being at peace, feeling anxious, or being worried about the illness or treatment, and whether their families and friends had been anxious or worried, required trust before they could be broached. They feared that the relationship between patients and HPs could otherwise be impaired, and that it might stress patients and relatives if such issues were raised at the wrong time The HPs regarded the question on whether patients felt at peace as causing the greatest problems. They reasoned that in their opinion hardly any patient can be at peace when receiving palliative care. They also said that they felt uncomfortable asking the question directly, as it was not formulated in the way they would like it to be	The HPs appreciated the focus on quality of life, as they considered it to be a relevant topic The HPs reported that multiple applications per case were rarely possible because of deteriorating health or because care periods are often too short for repetition. They assumed that this meant developments would not be identified when using this outcome measure. Some HPs felt it sounded like they were fishing for compliments when they asked patients if they thought ‘*the palliative care team is making a difference to how things are going*’ and expected no objective answers, for as long as they were providing care to the patients	The HPs valued the focus on the relatives when measuring outcomes, as they reckoned their needs are a relevant aspect of successful care. They also thought it made sense that the ZBI requires relatives to reflect on their own situation Nevertheless, the SOPC teams rejected use of the measure in practice. They told us that relatives had been outraged by questions on whether providing care had caused them to lose control of their lives, as they felt caring is a natural duty that they wanted to fulfil Furthermore, HPs thought using the measure was unsuitable because family caregivers’ answers could burden the patients, e.g. when they are asked whether the patient affected ‘*relationships with other family members or friends in a negative way*’ They also criticized the measure for not providing the differentiated feedback that would be helpful in practical work
Adaptation	We established the parallel use of self-reporting (written and oral) and proxy-reporting, whereby self-report was preferred. We further added the possibility to integrate topics into general conversation We avoided arranging predefined times, and suggested addressing the main problems/concerns and symptoms during the first assessment, and the other topics as soon as possible. The decision on what was appropriate was always made by the HPs	We changed the application from mandatory in all cases to voluntary, and for use when SOPC-team members considered it to be suitable We additionally integrated IPOS VoC into regular postal evaluations, which is another part of the ELSAH set of measures and will be described in another publication	We removed ZBI-7 from our set of measures. Instead, we developed and implemented a questionnaire for relatives that was based on the IPOS VoC patients’ version

*HP* health professionals

## Discussion

### Main findings

For the feasible, acceptable and appropriate integration of patient-reported and caregiver-reported outcome measures into the daily care routines of SOPC, the burden of its use on patients and relatives must be kept to a minimum. Furthermore, the usefulness of the measures must be clearly explained, care must be taken when broaching sensitive subjects, and administration should be manageable.

### Comparison of findings with those reported in the literature

Avoiding burden on patients and relatives was a major concern for SOPC-team members in our study. It is also an issue that is addressed in another study and reflects a common assumption in society that research into palliative care can be burdensome [38]. But it is preferable to permit patients and relatives to participate in care design, in research, and in quality improvement [39]. Evidence exists that both severely ill patients and their relatives are able to express their opinions on the quality of care [40]. Patients and relatives generally appreciate the chance to participate, provided they are not overburdened by their health condition or the demands of the study [39]. Kane et al. found that comorbidities complicated the use of IPOS in an inpatient palliative care setting, but did not necessarily overburden patients [41]. Creating a research culture through early communication and the request to participate in research can reduce the stress caused by deteriorating health [38]. Analogously, it is safe to assume that early explaining to patients and relatives how and why measures are used can help reduce stress.

Pinto et al. also found that health professionals fear that deteriorating symptoms may mask any improvement. They further fear that financing could depend on results [13]. Training and better understanding may reduce such fears, discourage health professionals from overprotection and gatekeeping, and promote their motivation to use PROMs by explaining how the results can be useful in practice [42]. Besides practical training in using the measures and explaining the rationale behind their application, it is therefore important to show their potential to improve quality on a micro, meso and macro level. At the same time, limitations should be addressed. Practical exercises and ongoing training would enable their use to be sustainable, and may also harmonize handling by different health professionals [19]. Howell et al. therefore suggest ongoing case-related education sessions and peer learning, combined with comprehensible reports on collected data [43]. A systematic literature review shows that most studies provide no guidance on how to react to problems [44].

In other studies, health professionals also have shown scepticism about the validity and reliability of the measures because of differences in the way they are handled in practice, e.g. by rewording in the interviewed self-report, or via proxy-reports [45, 46]. Although independent self-reports are preferable, it is also reasonable to interview them when it is necessary to reduce burden, or when patients are unable to participate [47]. Clapham et al. found that the incidence of self-reported symptom distress depends on the disease, and the urgency of needs, and that it is more common in an outpatient setting than an inpatient setting [48]. This reinforces our view that self-report in outpatient settings is feasible. As we found that the parallel use of self- and proxy-reporting can lead to interesting results in practical care, it should also be considered.

Furthermore, by demonstrating empathy when broaching sensitive topics, health professionals can reduce burden and scepticism. A predefined framework ensures comparability and orientation, but a successful SOPC approach requires flexibility and the ability to adapt to individual needs and situations [12]. Health professionals in other studies also feared that the use of PROMs may reduce the quality of relationships, but they also found that PROMs can result in open conversations and help patients raise topics of personal relevance [46].

As fixed time points caused feasibility problems, we decided to assess each topic upon inclusion in care, and to review them regularly when situations change. More guidance on when to use outcome measures may support health professionals and further improve comparability across services. An international expert consensus workshop therefore recommended the use of the ‘Phase of Illness’ to standardize time points for data collection, but it also emphasized that exceptions should be possible, depending on the patient's situation [49]. Bausewein et al. also said guidance can promote manageable use [50].

The need to document outcome measurements electronically is obvious. However, the question of what is a successful implementation also arises. Indeed health professionals in other settings also appreciate electronic documentation [42], and possibly even more so in an outpatient setting. In contrast to the inpatient setting, travelling to patients’ homes, and taking along paper involves greater administrative effort. A current review on the use of PROMs in oncology has identified lack of time as a barrier to use [51]. In outpatient palliative care, the time available for home visits is limited, and as health professionals meet patients less frequently, they must react immediately or wait until the next home visit.

According to a systematic review, having a coordinator in the team that is responsible for all implementation processes can facilitate successful implementation [6]. This aspect did not emerge in our study because the SOPC-team leaders automatically assumed the role of facilitator. When several SOPC teams are implementing measurements in parallel, it can be assumed that overarching coordination facilitates implementation.

The OACC suite of measures has been used in various studies. Similar to the feedback on IPOS in our study, health professionals in the inpatient setting struggled most with questions on psychosocial and family issues. Nevertheless, missing values decreased over the course of time [19]. We would expect ongoing training and familiarisation to improve the situation further.

Whereas the use of ZBI in our study was considered inappropriate and burdensome, another study found it to be appropriate [13]. This may be because our German translation had not been validated for use in palliative care. This has now been carried out for another study, and indeed translation problems were evident in the first version, and the measure was only recommended for use after translation adjustments [52]. As shown in feedback from our participants, Seibl-Leven et al. assumed the ZBI would lead to conflicting emotions and problems with loyalty. This is because relatives are asked to describe the burden caused by their ill relatives [53], which may be more relevant when relatives play a central role in the provision of care in an outpatient setting. Replacing the ZBI still seems to make sense. In the meantime, the OACC recommends the additional use of two questions for caregivers that are similar to the version we developed based on IPOS VoC [47].

### Strengths and limitations

In this study, we examined feedback from health professionals, but did not directly seek feedback from patients and caregivers. Health professionals may not have accurately reflected patients' attitudes, but their views on how patients might feel are a first approximation.

The study is limited by our gaining feedback from health professionals without observing them in action. Participant observations could have provided the opportunity to obtain practical insights [54].

As revisions had already been made when later teams were included, the intensity of collaboration and feedback from health professionals from different teams varied. To broaden our findings, we purposively sampled SOPC teams based on team location and documentation methods, and thus incorporated a variety of working conditions, attitudes, team structures and contexts. Although only a sample of SOPC team members participated in the focus groups, additional field notes meant all health professionals had opportunities to provide feedback. To get a deeper understanding of health professionals’ views, we triangulated our field notes in with focus groups. We conducted the focus groups in two SOPC teams, but did not combine members from different teams, although this might have made the discussion more diverse [32].

The Professional Association of Specialised Palliative Homecare in Hesse represents all SOPC teams in Hesse and is at the same time a research partner in this study. This may have biased our research, but the fact that we focussed on real-world implementation that will continue after the end of the study may have motivated health professionals to promote integration and provide honest feedback.

### Implications

Some of the aspects we identified were similar to those in studies in other settings. However, we also uncovered problems relating to the greater administrative effort and involvement of relatives in outpatient settings. This may help others avoid the difficulties we faced in our study. Furthermore, the collaboration between researchers and practitioners helped foster mutual understanding and is in our view to be recommended in other settings. Our findings are transferable to similar outpatient settings, but may be of limited use in SOPC care for children, as collaboration between health professionals, child patients and their families differs from the care of adults [55].

In routine specialised outpatient palliative care, patient- and caregiver-reported outcome measures provide a good basis from which to strengthen patients’ and relatives’ impact on care. Data collected using the described measures has not yet been analysed statistically, so testing on a larger sample was still pending at the time of this study. Routine data collection should also include the use of further regular, detailed surveys of patients and relatives receiving care, as well as surveys of bereaved relatives. In a further step, we will present a comprehensive concept on how to improve the quality of care in SOPC, which will build on the results described here [10].

Over the short term, implementation requires time and resources for training, integration into documentation systems and technical equipment, but additional work on data collection, administration, support and ongoing training is also necessary over the long term [7, 44, 56]. Benze et al. found high adherence of patients with advanced cancer in the use of a smartphone application in the outpatient setting [57]. More research is needed on how electronic PROM can be integrated into the outpatient palliative care setting through the use of, for example, web-based tools. Additional financial support is required to expand the use of PROMs in SOPC.

## Conclusions

The feasible, acceptable and appropriate integration of patient and caregiver outcome measures into daily care routines encourages their use. In this study, we found that although reservations about their implementation in a SOPC setting exist, appropriate adjustments can ensure their application in everyday care. For integration to be feasible, acceptable and appropriate, the burden on patients and relatives must be kept to a minimum, participants must understand the usefulness of the measures, empathy is required when exploring sensitive issues, and administration must be manageable. Implementation of the measures requires resources, especially for practical training, explaining the usefulness of the measures, designing manageable processes that include integration into electronic documentation systems, and for ongoing evaluation and support.

## Supplementary Information

**Additional file 1: A**. Focus group topic guide (translated into English). **B**. COREQ (COnsolidated criteria for REporting Qualitative research) Checklist.

## Data Availability

As the data may provide enough information to allow teams or participants to be identified, no original data will be published. The focus group topic guide and COREQ checklist [58] are provided as supplemental materials: Additional file 1.pdf. A. Focus group topic guide (translated into English) B. COREQ (COnsolidated criteria for REporting Qualitative research) Checklist

## Abbreviations

COREQ: Consolidated criteria for reporting qualitative research
DRKS: German Clinical Trials Register (‚Deutsches Register Klinischer Studien ‘)
CFIR: Consolidated Framework for Implementation Research
EDS: Electronic documentation systems
ELSAH: Evaluation of Specialised Outpatient Palliative Care by taking the example of Hesse
HP: Health professional
IPOS: Integrated Palliative Outcome Scale
IPOS VoC: IPOS Views on Care
OACC: Outcome Assessment and Complexity Collaborative
PROM: Patient reported outcome measure
SD: Standard deviation
SOPC: Specialised outpatient palliative care
ZBI: Zarit Burden Interview
ZBI-7: 7 Item Short-form Version of Zarit Burden Interview
