# Supplementary material for: Integrating patient- and caregiver-reported outcome measures into the daily care routines of specialised outpatient palliative care: a qualitative study (ELSAH) on feasibility, acceptability and appropriateness
Source: BMC Palliat Care. 2022 May 2;21:60. doi: 10.1186/s12904-022-00944-1 (PMC9063228; doi:10.1186/s12904-022-00944-1)
Supplement: Supplementary file 1 — Additional file 1: A. Focus group topic guide (translated into English). B. COREQ (COnsolidated criteria for REporting Qualitative research) Checklist. [file 12904_2022_944_MOESM1_ESM.pdf]

Additional file 1

Article: Integrating patient- and caregiver-reported outcome measures into the daily care routines of specialised outpatient palliative care: A qualitative study (ELSAH) on feasibility and practicality

Authors: Hannah Seipp, Jörg Haasenritter, Michaela Hach, Dorothée Becker, Lisa-R. Ulrich, Dania Schütze, Jennifer Engler, Cornelia Plöger, Stefan Bösner, Katrin Kuss

A. Focus group topic guide (translated into English)

- Please describe your experiences with the measures in general terms (applicability, comprehensibility, scope, content).
- What do you currently think about
    - … applicability?
    - … comprehensibility?
    - … scope?
    - … content?
- How do you incorporate the outcome measures into your daily work?
- Where do you see any room for improvement?
- What feedback have you received from users of the outcome measures?
    - Health professionals' views
    - Patients' views
    - Relatives' views
- Have you received any feedback on individual elements of the outcome measures?
- Do you have any suggestions/wishes concerning their further use ?

B. COREQ (COnsolidated criteria for REporting Qualitative research) Checklist

Item No.	Guide question/description	Answer (Reported in Section)
Domain 1: **Research team and reflexivity**		
Personal Characteristics		
1. Interviewer/facilitator	Which author/s conducted the interview or focus group?	HS, KK, and JH conducted the focus groups, meetings, and answered telephone calls and e-mails (Design)
2. Credentials	What were the researcher's credentials? E.g. PhD, MD	Master's degree, PhD, professor or manager of the Professional Association of Specialised Palliative Homecare in Hesse (Authors' information)
3. Occupation	What was their occupation at the time of the study?	Researchers at the universities of Marburg and Frankfurt, management of

		the Professional Association of Specialised Palliative Homecare in Hesse (Authors' information)
4. Gender	Was the researcher male or female?	HS, MH, DB, DS, JE, CP, KK are female, and JH, SB male
5. Experience and training	What experience or training did the researcher have?	Designing and conducting previous research in general practice and palliative care
Relationship with participants		
6. Relationship established	Was a relationship established prior to study commencement?	Yes, a relationship was established in a previous phase of the study (sampling)
7. Participant knowledge of the interviewer	What did the participants know about the researcher? e.g. personal goals, reasons for doing the research	The participants knew the researchers' occupations and the overall aim of the study
8. Interviewer characteristics	What characteristics were reported about the interviewer/facilitator? e.g. Bias, assumptions, reasons and interests in the research topic	The Professional Association of Specialised Palliative Homecare in Hesse represents all SOPC teams in Hesse and was a research partner in this study (strengths and limitations)
Domain 2: **study design**		
Theoretical framework		
9. Methodological orientation and Theory	What methodological orientation was stated to underpin the study?	We used a multi-method qualitative design and qualitative content analysis (aim, analysis)
Participant selection		
10. Sampling	How were participants selected? e.g. purposive, convenience, consecutive, snowball	SOPC teams were purposively sampled (sampling)
11. Method of approach	How were participants approached? e.g. face-to-face, telephone, mail, email	We approached SOPC-team leaders face-to-face and per email.
12. Sample size	How many participants were in the study?	Five SOPC teams participated in the pilot run and 14 team members in the focus groups (sample)
13. Non-participation	How many people refused to participate or dropped out? Reasons?	All invited SOPC teams participated (sample)
Setting		
14. Setting of data collection	Where was the data collected? e.g. home, clinic, workplace	The multi-method qualitative design meant data was collected in various ways (design)
15. Presence of non-participants	Was anyone else present besides the participants and researchers?	No one else took part in the focus groups

16. Description of sample	What are the important characteristics of the sample? e.g. demographic data, date	The important characteristics are presented in the main text (sample)
Data collection		
17. Interview guide	Were questions, prompts, guides provided by the authors? Was it pilot tested?	The focus group topic guide is provided as supplemental material
18. Repeat interviews	Were repeat interviews carried out? If yes, how many?	Not applicable
19. Audio/visual recording	Did the research use audio or visual recording to collect the data?	We audio recorded the focus group (design)
20. Field notes	Were field notes made during and/or after the interview or focus group?	We collected field notes during focus groups, meetings and conversations (design)
21. Duration	What was the duration of the interviews or focus group?	Information is provided on how long they lasted (sample)
22. Data saturation	Was data saturation discussed?	Repeated requests and numerous opportunities enabled the views of all participants to be included (strengths and limitations)
23. Transcripts returned	Were transcripts returned to participants for comment and/or correction?	No
Domain 3: **analysis and findings**		
Data analysis		
24. Number of data coders	How many data coders coded the data?	HS coded the data (analysis)
25. Description of the coding tree	Did authors provide a description of the coding tree?	No
26. Derivation of themes	Were themes identified in advance or derived from the data?	We took into account the themes identified during data collection and added some emerging topics (analysis)
27. Software	What software, if applicable, was used to manage the data?	We use MAXQDA (analysis)
28. Participant checking Reporting	Did participants provide feedback on the findings?	Participants received feedback through the iterative process of feedback and adaptation (design)
Reporting		
29. Quotations presented	Were participant quotations presented to illustrate the themes / findings? Was each quotation identified? e.g. participant number	Yes (results)
30. Data and findings consistent	Was there consistency between the data presented and the findings?	Yes (results)

31. Clarity of major themes	Were major themes clearly presented in the findings?	Yes (results)
32. Clarity of minor themes	Is there a description of diverse cases or discussion of minor themes?	Yes (results)

Based on: Tong A, Sainsbury P, Craig J. Consolidated criteria for reporting qualitative research (COREQ): A 32-item checklist for interviews and focus groups. International Journal for Quality in Health Care. 2007;19:349–57. doi:10.1093/intqhc/mzm042.
